# Harnessing the Interaction Continuum for Subtle Assisted Living

**DOI:** 10.3390/s120709829

**Published:** 2012-07-23

**Authors:** Manuel García-Herranz, Fernando Olivera, Pablo Haya, Xavier Alamán

**Affiliations:** 1 Escuela Politécnica Superior, Universidad Autónoma de Madrid, Madrid 28049, Spain; 2 Ingeniería Mecatrónica, Universidad Politécnica de Zacatecas, Fresnillo (Zac) 99059, Mexico; E-Mail:folivera@upz.edu.mx; 3 Instituto de Ingeniería del Conocimiento, Universidad Autónoma de Madrid, Madrid 28049, Spain; E-Mail: pablo.haya@iic.uam.es; 4 Escuela Politécnica Superior, Universidad Autónoma de Madrid, Madrid 28049, Spain; E-Mail: xavier.alaman@uam.es

**Keywords:** subtle interaction, ambient assisted living, ambient intelligence, awareness, cognitive disabilities

## Abstract

People interact with each other in many levels of attention, intention and meaning. This Interaction Continuum is used daily to deal with different contexts, adapting the interaction to communication needs and available resources. Nevertheless, computer-supported interaction has mainly focused on the most direct, explicit and intrusive types of human to human Interaction such as phone calls, emails, or video conferences. This paper presents the results of exploring and exploiting the potentials of undemanding interaction mechanisms, paying special attention to subtle communication and background interaction. As we argue the benefits of this type of interaction for people with special needs, we present a theoretical framework to define it and propose a proof of concept based on Augmented Objects and a color codification mechanism. Finally, we evaluate and analyze the strengths and limitations of such approach with people with cognitive disabilities.

## 1. Introduction

Nowadays, families in western societies are more decentralized and gather around smaller cores. Life expectancy has grown, and birth rate has decreased, leading to an aging society with an increasing number of elders living alone. Similarly, helped by social programs and a global concern of integration and equal opportunities, people with special needs have now a better chance to live on their own. However, these two sensitive examples of independent living can easily see their independence turned into an isolation in which daily activities become difficult tasks to overcome and health issues and accidents hard to prevent and detect. Therefore, strongly relying on a small social fabric, these collectives are many times forced to trade their independence to avoid solitude.

Ambient Assisted Living is a field of research looking for technologies to support and aid people in their homes. While many of its efforts have focused on designing intelligent objects or systems to directly assist users [1], there is a growing concern on the importance of creating technologies to strengthen social ties. This social dimension of Ambient Assisted Living serves the double purpose of easing assistance—by leveraging communication—while breaking isolation—by empowering a sense of community.

Most of the vast set of existing communication technologies—such as phone calls, chats, social networks, video conferences or virtual worlds—offer rich but intrusive and demanding communication channels, best suited for punctual interactions than for creating a seamless atmosphere of connectedness.

Short message services—such as SMS, WhatsApp or Viber—offer, on the other hand, limited communication channels to the benefit of a more unobtrusive and pervasive communication experience. This type of *soft* or *cheap* (in terms of effort) communication is an invaluable tool to strengthen social ties, leading many times to more richer interactions such as phone calls or visits. We refer to this type of interaction in which “less is more” as *subtle interaction*. An interaction in which special attention is paid to balancing communication and intrusion.

Technologically, different disciplines already offer a robust background to build upon. New media for distributed subtle interaction can take advantage of the many contributions of both Augmented Objects and Tangible User Interfaces (TUI) to, in Ishii and Ulmer words, “augment the real physical world by coupling digital information to everyday objects and environments”[2]. The possibilities of TUI and Augmented Objects are vast and varied, from enhancing common objects [3], creating new ones [4] or projecting information onto existing ones [5,6]. In this sense, several studies have addressed the challenges of prototyping smart objects [7], giving some useful design heuristics and classifications [8] and developing platforms for prototyping them [9].

Human Computer Interaction (HCI), in addition, has explored subtle interaction in peripheral displays [10] as well as studied the different phases of interaction [11] and even established a framework for interaction phases [12].

Following this technological evolution, within Ambient Assisted Living, some projects have already focused on subtle interaction too, to provide awareness [13], enhance affective ties [14] or trigger and benefit from emotional contexts [15]. However, as we lack a global framework for human communication and interaction (HHI), most of these pioneering efforts focus on specific needs and fail to provide scalable solutions that can be replicated to traverse from awareness to interaction, combine HCI and HHI needs or extend communication to social groups or larger scenarios.

Following the work started in [16,17], this paper presents in Section 2 an extension of Ju and Leifer's Interaction framework [18] (defined along the axis of Attentional Demand *vs.* Initiative) with a Communication framework (defined along the axis of Traffic *vs.* Reaction), inspired by Shannon's definition of information [19]. Using this framework, we design and develop an augmented object platform for subtle interaction in Ambient Assisted Living, introduced in Section 3. In Section 3.2 two natural languages are defined based on natural associations of symbols and colors respectively, additionally, a simple experiment is presented to test short-term memory of small lists of person-color associations. Finally, in Section 4, we present the results of an experiment to test the naturalness of the color language defined in Section 3.2 as well as to measure the impact of adding dimensions to simple languages in groups with Down Syndrome.

## 2. A Theoretical Framework for Subtle Interaction

**Figure 1.**
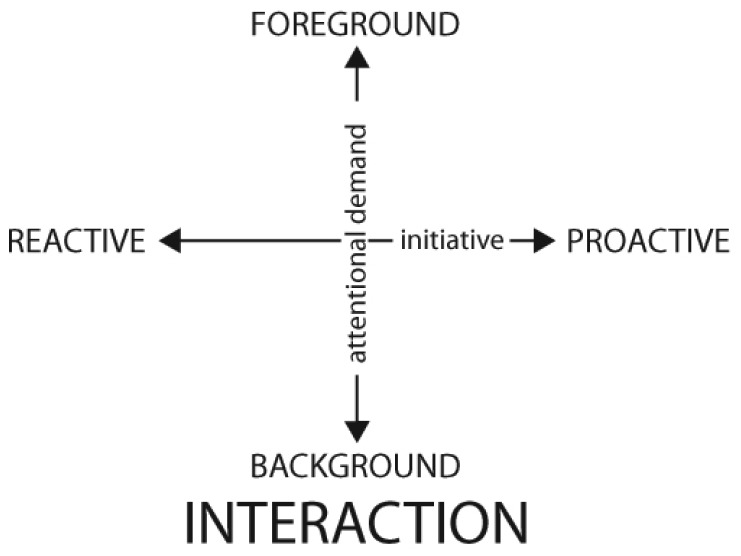
Interaction classification along the axis of attentional demand and initiative according to Ju and Leifer [18].

According to Ju and Leifer [18] the space of possible interactions can be divided along the axis of attentional demand and initiative (see Figure 1); that is, how much attention is required in the interaction and who initiated it.

This framework is especially suited for HCI and, regarding technology, since different communication devices are designed to suit the needs of different types of interaction, we can use this framework to classify them.

Focal (foreground) devices, such as PCs, TVs or phones, are designed to support long-term interactions with detailed information and therefore usually require most of the user's attention. Lateral (background) devices, such as the indicating lights of a car, a beeper or the battery indicator of a phone are designed to support short-term interactions with limited information needing few of the user's attention.

Traditionally, lateral interaction has been used to communicate limited and predefined information, in HCI from a system to the user (e.g., the small icon or light showing the battery level of the device) or, in rare cases, in HHI, from person to person (e.g., the indicating lights of a car to advise other drivers before turning). However, in direct interactions people use lateral communication in more flexible and powerful ways not only to transmit information but also, and most importantly, to trigger different reactions.

In order to create technologies to support this kind of interaction we need to incorporate—and understand—this empathic dimension to our frameworks.

Shannon defined entropy as a measure of how much information is carried in a message [19]. However, while he stated that *frequently the messages have meaning he discarded these semantic aspects of communication as irrelevant to the engineering problem*. While this is perfectly true from the perspective of reproducing at one point the message selected in another point, it is not so when considering the reactions the message triggers. Similar messages, or even the exact same message, trigger different reactions depending on context. These reactions can be categorized (from their informational nature to the different feelings they trigger) and, most probably, objectively measured at least in terms of neuronal interactions or brain activity (either considering all neurons or activated brain regions equally important or not). While this objective measure falls way beyond the scope of this paper, a subjective measure suffices to understand the different reactions a simple “yes, I do” can trigger in different contexts.

Thus, theoretically, we need to define communication also in terms of reaction transactions to, technologically, understand how to turn the limited means of lateral interaction into flexible channels to communicate feelings and emotions.

From here on, in order to ease the reading, we will refer to what we have previously called *reactions* (to differentiate them from Shannon's information) as *information*, since they include the whole set of pieces that, in Shannon's words, are “produced” by a particular message. On the other hand we will refer to *information*, in the sense stated by Shanon, as *traffic*, since it represents the number of minimum bits with which the message can be transmitted.

**Figure 2.**
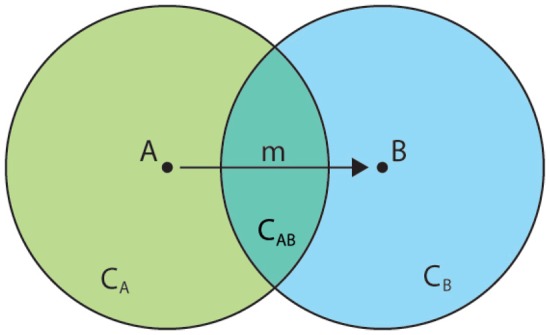
Context-aware Communication schema considering sender (*A*), receiver (*B*), message (*m*), and the overlapping contexts of *A* and *B*.

In the classical schema (see Figure 2), *A* (the sender) sends a message to *B* (the receiver) using some kind of media but, in terms of information, it is vital to consider the context-aware dimension of communication. Consequently, *C_A_* and *C_B_* are *A* and *B*'s respective contexts, which may overlap in some common context *C_AB_. C_AB_* includes common knowledge such as *X* when *A knows X* and *B knows X*, but also, and most importantly, interpersonal knowledge such as *B knows X* when *A knows B knows X* or *A knows B knows X* when *B knows A knows B knows X*. In fact, our empathic nature allows us to extend our interpersonal knowledge with more or less accurate guesses and deductions that can lead to different perceptions of *C_AB_ i.e., C_AB_* ≠ *C_BA_* or ∃*x* ∈ *C_AB_*: *x* ȩ *C_B_*, mother of uncountable misunderstandings and losses throughout history. For the sake of simplicity we will ignore this phenomenon and think of *C_AB_* in a less strict way.

When *A* creates a message for *B*, it does so from her context *C_A_* but shaping through what she knows of *B* (*i.e., C_AB_*) so it can ideally be understood by *B* in her context *C_B_* as *A* originally intended. *B*, on her side, interprets the message through her context *C_B_* but, knowing it comes from *A*, uses the common context *C_BA_* to try to understand what *A* meant in her context *C_A_* (see Figure 2). Despite its apparent complexity, this interpretation is most of the times automatic, a reflex of communication as an empathic process, enriching and shaping the message with the common context of sender and receiver for a better understanding. Being careful with words, using supporting sentences or finishing other's ongoing sentences are some of the many examples found in daily life that define human communication as a context-aware empathic process.

This phenomenon leads to the situation in which a message sent to or created by two different persons result in different information. Formally:

(1) $$i=f_{B}(m,A,C_{B})$$

where *i* is the information received by *B, f_B_* is *B*'s interpretation function, *m* is the message sent by *A, A* is the sender and *C_B_* is *B*'s context, where *C_BA_* plays an extremely important role.

**Figure 3.**
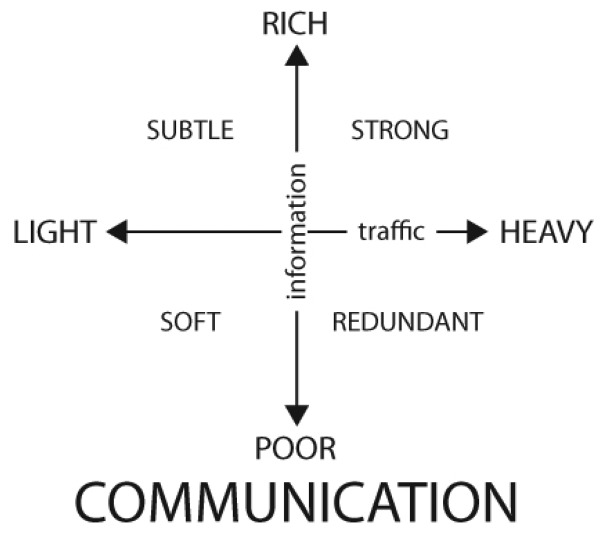
Our proposal for a communication classification along the axis of information and traffic. Traffic refers to the size of the message that is sent. The information axis considers the impact of that message in the receiver.

Thus, considering message and information as two different variables, we classify communication in a two axis continuum according to the sizes of the generated communication traffic (*i.e., m*) and the information—the complete set of reactions—extracted by the receiver (*i.e., i*) (see Figure 3).

In this schema, considering *r* = *i/m* the size ratio between information and traffic, we can find *r* ≃ 1 (either with high or low values of *i* and *m* in strong and soft communications respectively), *r* < 1 for heavy traffic leading to poor information (in redundant communications) or *r* > 1 for light traffic leading to rich information (gluing pieces of existing context with the message to create a richer one in subtle communication).

Human interaction traverses through the interaction and communication continua of Figure 1 and Figure 3, reacting to information and communication needs, from a subtle move to reject more coffee to starting a philosophical debate in the same coffee place. Within this double continuum, we focus on the binomial composed by subtle communication (see Figure 3) and background interaction (see Figure 1) to define subtle interaction and design communication technologies to support it remotely.

### 2.1. Computer Supported Subtle Interaction

Subtle interaction builds upon context through secondary channels. This means that most information is not present in the message but in the common history and knowledge of both sender and receiver. It relies on empathy and mutual understanding to create rich communication channels from simple background messages: A wink carrying almost no information uses context to create a dialog, something new and bigger such as “I love you” or “be careful”.

However, while in face to face interaction sender and context are a natural part of the scene, a remote mechanism to support subtle interaction requires to communicate them somehow along with the message. In other words, the generated traffic *x* must codify the message *m*, the sender *A* and a piece of context *C* so it can be decoded on arrival.

(2) x=c(m,A,C)

(3) $$c^{-1}(x)=\{m,A,C\}$$

Thus, the interpretation process of Equation (1) is transformed into a decodification and interpretation process:

(4) $$f_{B}(c^{-1}(x))=f_{B}(m,A,C)=i$$

As the unobtrusiveness of subtle interaction relies strongly in its simplicity, it is vital to reduce the complexity of the decodification function *c*^−1^(*x*) to the minimum so the overall process remains as natural and automatic as possible.

## 3. An Application Case for Subtle Interaction

As subtle interaction is based on context (e.g., who, where, when or about what), a tool to support long distance subtle interaction needs to communicate context without significantly increasing the communication traffic. In our proposal we rely on distributing the interface among diverse smart objects so they can be used to implicitly codify part of the context, reducing the amount of traffic that has to be transmitted in the message. As a sticker with the words “Eat me” takes advantage of the physical location in which it is placed to implicitly complete the message, we use a distributed interface to allow an implicit specification of the most basic “what is the message referring to” piece of context. Therefore, part of the message (a pointer to the context *C* to which the sender is referring) is implicitly codified in the object *O* chosen to show the message, leaving *x* to codify just the sender's identity (*A*) and the message itself (*m*).

In its most basic form, the message is used to codify just the sender's identity (*A*), reducing *m* to a limited “*A winks*” resulting in a simple message *O*(*x*) = “*A winks through object O*”.

As codifying mechanisms we have explored the use of symbols and colors as they offer the simplest forms of visual recognition. Being aware that approximately 6% of men (and 0.03% of women) suffer color blindness of some degree [20], this proposal must be considered as a significant example to show the potentials of subtle interaction, rather than as a technological breakthrough. However, a similar approach can be applied to other types of codes, such as using icons, sounds, vibration patterns or any combination of them that remains to be further explored. Nevertheless, the main idea of communicating message *m* and sender *A* in the code while obtaining a pointer to the referred context implicitly from the object in which the code is shown remains the same.

We believe that such a limited communication mechanism for subtle interaction can trigger strong communication/affection channels in an unobtrusive and easy way.

This communication paradigm is of special significance in Ambient Assisted Living since it provides an integrated platform to bring users virtually closer to their relatives, opening an unobtrusive but empathic complement to phones, visits or letters. Furthermore, this type of setup allows for implicit interaction by connecting distant spaces to leave automatic digital footprints in their counterparts (e.g., showing that the grandson opened his fridge by making grandmother's glow with his color).

This distributed simple platform can be also used in Intelligent Environments to provide guidance and awareness, from directing attention to food that is going to be outdated to reminding to take pills, to do the laundry or to follow a particular diet through the very objects involved in the action.

This is, in turn, the result of applying the theoretical framework presented above: a multi-purpose lateral device allowing for subtle interaction. As stated by José *et al.* [21], we focus on *reusable elements that are applications in the sense that they provide some useful service directly to users, but at the same time have the ability to become building blocks to the creation of multiple systems*.

Following the guidelines stated in [17] we have developed an embeddable interface using RGB LED buttons to obtain a simple low-resolution multi-touch screen. While these interfaces can be built with different shapes, to adapt them to the particulars of different objects, our prototype consists in a 4 × 4 matrix, a purposely small resolution big enough to code the most basic symbols.

Choosing RGB LED buttons instead of just RGB LEDs gives the interface a bidirectional dimension which is both used to allow feedback/acknowledge as well as to traverse to richer levels of interaction/information in an attempt to replicate the continuum of natural communication. In this sense, the interface needs to differentiate between attempts to communicate with the sender (e.g., acknowledging a message) from those with itself (e.g., requesting more detail in the information displayed).

Having 16 buttons on our interface we could have chosen to assign different functions to each of them. Instead we decided to build upon the basics of gathering to design an interface in which time defines purpose: actions are fast, choices or searches slower. Thus, we speed up interaction when the action is clear while forcing to spend a little longer for getting clarifications. Therefore, the two basic types of interaction we propose are:

- Short press. To interact at the current communication level: acknowledge the message or send feedback.
- Long press. To traverse to a richer information/choice level: requesting more detail on the information or more options to choose from.

**Figure 4.**
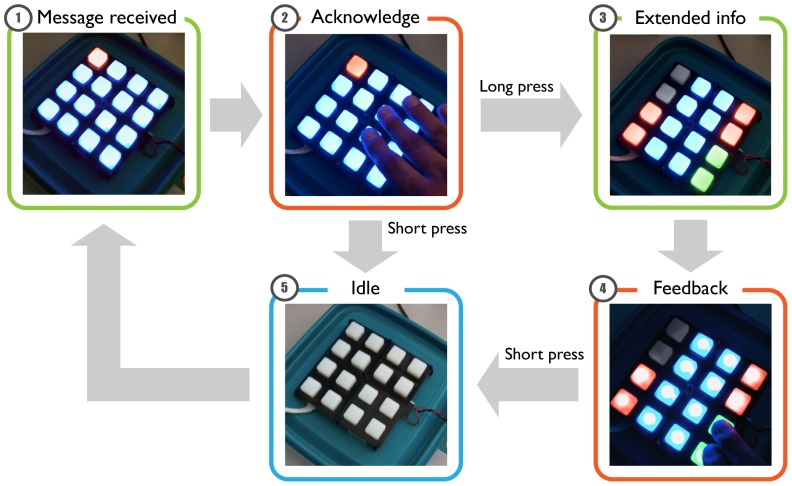
Distant Human to Human Communication on an augmented Tupperware™.

**Figure 5.**
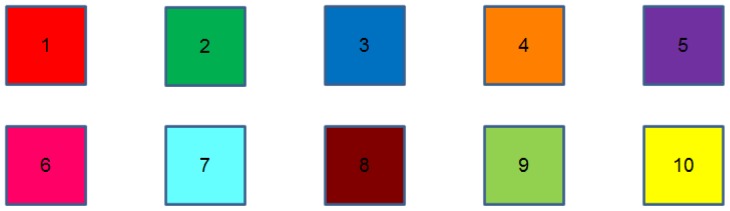
Available colors to establish person-color associations.

As an example of HHI, when a message is received (see Figure 4, 1) the LED matrix glows in the sender's associated color (e.g., blue) while one of the corner LEDs (red in this case) uses a green-yellow-red code to show the sender's opinion. At this point (see Figure 4, 2), the user can either quickly acknowledge the message with a short press, reseting the device to its idle state (see Figure 4, 5) or press longer to request extended info (see Figure 4, 3). In our example the device now shows more granularity in the recommendation (2 red LEDs from a maximum of four), along with an improvised two red-lit/two green-lit LEDs on the bottom to allow for positive or negative feedback to the sender (see Figure 4, 4). The rest of the interface would retain the sender's color and a short press on them will serve the same purpose as the acknowledge stated before.

In an example for HCI, the interface displays a red-yellow-green color code to express the level of urgency to take an action, such as to decide what food has to be consumed first. In this case, a short press will discard the information, as an acknowledge to the system that the information has been received. A long button press, on the other hand, as a request for more information, changes the display to show as many illuminated LEDs in the matrix as days left until the expiration date.

This process, while needing some initial explanation builds upon natural concepts that can be universally applied to traverse interaction.

### 3.1. Person-Color Associations

However, looking at the simplest scenario of HHI in which the interface only shows the sender's color and no feedback is allowed or wanted, we need to determine whether the person-color association is powerful enough to allow identification of senders in small social networks.

Person-color associations are widely used in many domains, from board games to napkin rings in order to define ownership. Nevertheless in most of these domains the other person is physically present to both stress the association and correct possible misunderstandings. Therefore the question of how many of this associations are we able to remember and how long these associations last remains still open.

In order to define a working basis we have conducted a series of experiments to define the gross boundaries of short term memory for person-color associations. These experiments have been conducted over 44 students between 19 and 22 years old of Universidad Politécnica of Zacatecas. From this 44 users, 8 were discarded due to execution problems.

Students were separated into two groups of the same size and were asked to write down a list of 10 persons of their closest social circle. To group 1, different colors (see Figure 5) were randomly assigned to each person of their lists, group 2 was given the list of colors so they could choose which color to assign to each person. Both groups were given 5 minutes to memorize their associations (group 2 had 5 minutes to both assign the colors and memorize them).

**Figure 6.**
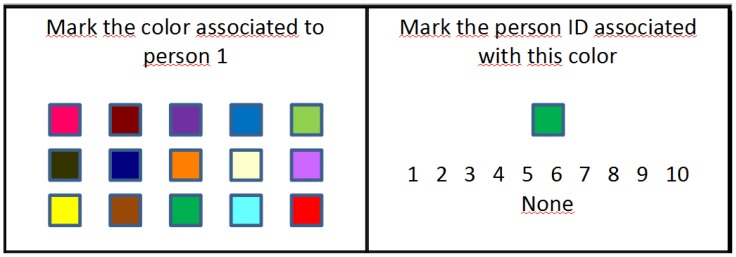
Examples of the question cards used in the person-color association study.

Each student was then given a list in which the names stated at the beginning of the experiment were randomly assigned to the numbers 1 to 10. This list was used as a guide during the test to respond to 24 questions (see Figure 6): 10 *which color correspond to person X?* and 14 *which person correspond to this color?*. In the former, there were 15 possible color answers (the original 10 colors plus 5 extra colors that had not appeared before). The latter included 4 questions about colors that had not appear before, being the possible answers the IDs of the 10 persons in the list plus an extra *none* answer.

This set of questions were repeated to both groups after a 30 minutes period of other non-related activities.

The results showed no statistical difference between both groups and both trials. Group 2 recognized correctly 8.5 associations (mean) with a standard deviation of 1.35.

An average of 8 person-color associations is a very good indicative to use this mechanism as an identity encoder for small social groups. Additionally, these results showed that over a short time period there is no difference between associations created by the subjects and associations forced by random. Further studies need to be conducted for long-time memory and to measure how letting users choose the colors affect the results.

### 3.2. Simple Languages for Subtle Interaction

Besides the person color associations to define the sender *A*, a limited display such as our 4 × 4 RGB LED matrix, plus the need for a natural decodification function, require to define simple languages to codify the message *m*. Focusing on colors and symbols we conducted a set of experiments to determine the natural associations they have with “urge” or “negativity”.

The experiments were conducted with 44 students between 19 and 22 years old of Universidad Politécnica de Zacatecas. Two comparisons were carried out, one with colors, the other with symbols, in which items were presented in pairs in order to make a pairwise comparison. Subjects had, therefore, to decide which symbol or color, hypothetically placed onto identical products in a fridge, signaled the product closer to expire.

For the color experiment we used red, yellow, green and blue to establish their natural order and distance. For the symbols experiment we used saltire (×), dash (-), cross (+), square (□), triangle (△) and circle (○).

In order to sort items, we computed the total score *S* as the number of times in which an item was considered as indicating the product closer to expire. Then, for each pair of items we compared the difference between their total scores as well the number of times each item was chosen against the other. The results are presented in Tables 1 and 2 in which columns and rows represent the items *I* (with their total scores in parentheses) and each cell *c_ij_* show the results of comparing item *I_i_* in row *i* with item *I_j_* in column *j* as *A/B* − *C*, where *A* = *S_i_* − *S_j_* is the difference between total scores, *B* is the number of times in which item *I_i_* is chosen against *I_j_* when compared directly and *C* is the number of times in which item *I_j_* is chosen against *I_i_* when compared directly. Therefore *B* + *C* = 44.

The differences have been compared using the *χ*^2^ test. The statistical significance of the difference is represented in Tables 1 and 2 by * for *p* < 0.1, ** for *p* < 0.05, *** for *p* < 0.001 and bold letters for no significance.

As we can see in Table 1 a clear difference can be established from *Red* to *Yellow* to *Green* being, as expected, almost equally distributed. However, there is no statistical difference between *Green* and *Blue*, which seem to be almost interchangeable for every purpose.

Within the symbols, as we can see in Table 2, there is a significant difference between the saltire (×) and the rest of the items, while the square (□), triangle (△) and circle (○) clearly lie at the other semantic end, being almost interchangeable. The dash (-) and cross (+) lie somewhere in the middle, with the dash (-) a little closer to the saltire (×) than the cross (+).

Therefore, according to the results we can define the following sequences, of different granularity, as being naturally sortable:

*Red Yellow Green* or *Red Yellow Blue*
*Red Green* or *Red Blue*
×- + □ or × - + △ or ×- + ○
× - □ or × - △ or × - ○
× □ or × △ or × ○

### 3.3. Application to Ambient Assisted Living

As stated before, we believe that Subtle Interaction, as a light way of background interaction, is particularly useful in Ambient Assisted Living, enhancing an easy to process and unobtrusive experience to provide guidance and strengthen the social fabric. That is, preserving independence while supporting in daily activities and breaking isolation.

**Figure 7.**
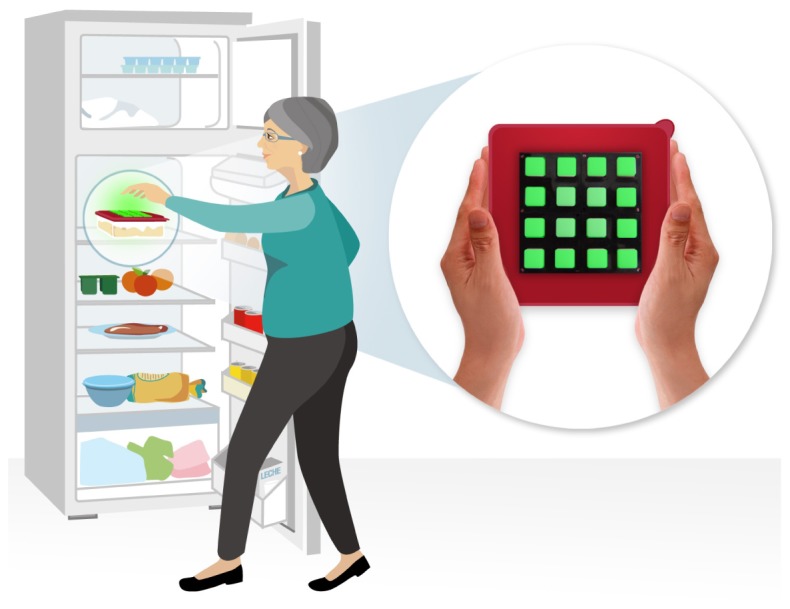
A prototype of an augmented Tupperware™ with the 4 × 4 button-RGB LED matrix integrated in its lid.

Although many collectives can be beneficiary of this technology, we assume that people with cognitive disabilities would show more difficulties in adopting it. Therefore, as a proof of concept, we focused on eating and cooking as a common problem among people with special needs living independently in order to test the simplicity of our approach and language definition. Taking advantage of the rich context that is generated around this issue we augmented Tupperwares™with the 4 × 4 button-RBG LED matrices described before (see Figure 7).

The Tupperware™ prototype provides a reduced but rich scenario in which, at opening the fridge, the user may find different lighting color or symbol codes indicating how close are some expiring dates or showing subtle messages from other people of their network such as reminders, suggestions, encouragement or plain empathy.

Nevertheless, while the simple languages presented in Section 3.2 have been defined through user studies, the cognitive limitations of particular groups such as people with Down Syndrome require to further test their simplicity within these groups. Additionally, having defined more than one language (*i.e.*, colors and symbols) it is possible to combine them in order to build a multidimensional language with a combinatorial number of possible messages and therefore more expression capabilities. However, especially when dealing with people with cognitively disabilities, it is important to understand how the extra complexity of multidimensional coding affects recognition.

## 4. Protoype Evaluation

### 4.1. Color and Multidimensional Codes for Assisted Living

In order to test the simplicity and limitations of the codes established in Section 3.2 as well the implications of multidimensional codes among people with cognitive disabilities we conducted a series of tests with these collectives.

Within these tests, the Tupperware™ prototype was configured to show only recommendations based on food expiring dates. In this scenario the meaning of the message is independent from the sender, since, as stated in Section 3.2, the green/yellow/red code as well as the symbol codes are defined as independent and socially accepted codes.

Besides, it is worth noting that the food scenario, as a real issue in independent living situations for people with cognitive disabilities, eases external validation of the results.

The experiment was conducted with 28 participants between 18 and 30 years old. The sample consisted of students of the Universidad Autónoma of Madrid in the first and second course of an employability project for cognitively impaired people. Their intellectual disabilities range from Down syndrome (primary trisomy and mosaicism) to Turner syndrome, cerebral paralysis and encephalopathy without specified etiology.

Their technological background is based on two courses taught at the first and second years of the project, respectively. Their programs contemplate the use of Internet and a text editor as indispensable tools to complete their training and widen the range of future possible employments.

We conducted the experiment based on a within-subjects design with two conditions, each condition considering a different coding scheme. Condition 1 (C1) was conducted showing only colors in our interface, while condition 2 (C2) used a combination of symbols and colors.

Participants were asked, after a brief explanation to ensure the task was properly understood, to interpret the meaning of the illuminated LED matrix of the prototype. This test was conducted twice with several weeks of separation and interchanging the condition, so that participants assigned to C1 in the first run where assigned to C2 in the second one and vice-versa.

**Figure 8.**
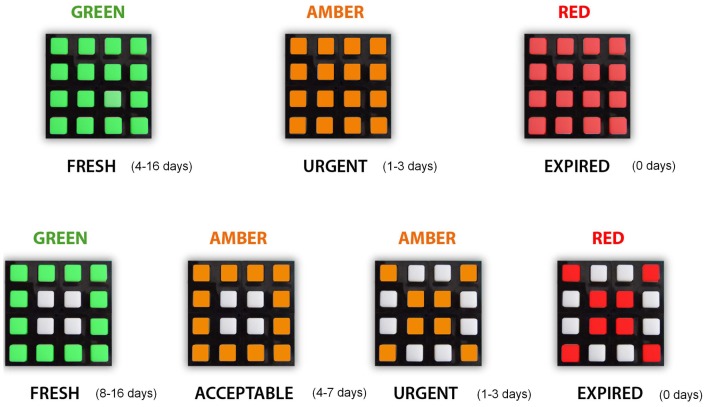
Code for both cases of study (Symbol association to concepts and days for expiration).

C1 was designed to codify food state in three “days to expire” categories: Fresh (between 16 and 4 days to expire), Urgent (between 3 and 1 days to expire) and Expired (expired product). The categories were codified in C1 using only colors: *Green* for Fresh, *Yellow* for Urgent, and *Red* for Expired (see Figure 8).

In C2 a new category Acceptable (between 7 and 4 to expire) was added while the Fresh category was narrowed to “between 16 and 8 days to expire”. Therefore C2 offered higher resolution for food life than C1. Categories were codified in C2 as a combination of color and symbols. To minimized complexity we used the color code of C1 and the simplest symbol code of Section 3.2 □ ×. In addition, no hierarchy was established between codes in such a way that a × symbol will always code a smaller number of days to expire than a □ symbol, regardless of their color. In the same way a *Red* color will codify a smaller number of days to expire than a *Yellow* color, smaller than a *Green* one, regardless of the symbols used. Therefore, the simplified multidimensional code of C2 uses *Green* □ for Fresh, *Yellow* □ for Acceptable, *Yellow* × for Urgent, and, finally, *Red* × for Expired (see Figure 8).

In both conditions participants were asked twelve times to guess the number of days to expiration coded in the message, therefore the finer granularity of C2 seemed, a-priori, an advantage over C1.

During the first six trials subjects had a printed copy of the code available to consult the correspondence between the name of the concepts and the range of days they represented (without any information about the color and symbols used to represent them). This printed copy was removed for the rest of the test, therefore, the first six trials have not been considered for statistical purposes.

In addition, after answering each question, participants were asked to validate their answers by requesting more information to the interface. This was done through the long press mechanism defined in Section 3, causing the interface to show as many illuminated LEDs as exact days were left to expiration. We asked the participants to write down the number of illuminated LEDs appearing in the interface to later compare it both with their interpretation of the color/symbol code and the real number of days left to expiration.

We have measured prediction performance using three different metrics:

1. The absolute difference between the answered concept and the correct one. This metric required mapping each concept to a numerical value. For the C1 the mapping was *Fresh* to 0, *Urgent* to 1, and *Expired* to 2, and for the C2 was *Fresh* to 0, *Acceptable* to 1, *Urgent* to 2, and *Expired* to 3. For instance, if the subject answered *Urgent* to a *Fresh* item, the absolute difference in C1 will be 2 while 3 in C2.
2. The absolute difference between the guessed number of days and the correct number of days to expiration.
3. A binary value representing whether the participant made a mistake in counting the number of illuminated LEDs when in the extended info stage.

## 5. Prototype Evaluation

### 5.1. Results and Discussion

In C1, 92% of the participants made a correct guess over all concepts, a huge number compared to just the 34.8% found in C2. It was observed that in C1, mistakes were only made by mixing Urgent with Expired (4 mistakes) and Expired with Urgent (3 mistakes) (see Table 3), while in C2 the most common error was to answer Acceptable in Urgent items (14 mistakes), followed by answering Urgent in Acceptable items (10 mistakes). Some subjects also mixed Expired with Urgent (4 mistakes) in C2 (see Table 4).

For statistically analyzing the absolute difference between the answered concept and the correct one, the results were normalized by the number of times each participant repeated the test (6 times). After normalizing, we performed a Kolmogorov-Smirnov normality test showing that the distribution of the normalized absolute differences was non-normal for both conditions (C1 *D*(25) = 0.534, *p* < 0.001 and C2 *D*(23) = 0.197, *p* < 0.05). Thus a Wilcoxon Signed Ranks Test was chosen as it is a non-parametric test well suited for within-subject designs. We found that participants made significantly more mistakes in guessing the correct concept under C2 (*M dn* = 0.17) than under C1 (*M dn* = 0, *Z*(20) = −3.09, *p* < 0.05, *r* = −0.48). The effect size indicates that this difference represents a large, and therefore substantive, effect.

A similar analysis was performed to analyze the absolute difference between guessed and correct days to expiration, finding, again, that data in both conditions were non-normal. However, in this case, a logarithmic transformation allowed us to obtain a normal distribution for both C1, *D*(25) = 0.154, *p* = 0.128 and C2, *D*(23) = 0.090, *p* = 0.200. After applying a paired-sample t-test to the logarithmic distributions, no statistical significant difference was found (*t*(19) = 1.053, *p* = 0.306) between the answers in C2 (*M* = 0.34, *SE* = 0.23) and those in C1 (*M* = 0.26, *SE* = 0.36).

Regarding the difference between counted days and number of illuminated LEDs in the extended info stage, both conditions show an 80% of correct answers.

These results show that many subjects encountered problems in identifying color/symbols combinations, suggesting that multidimensionality in the code poses a great challenge to people with cognitive disabilities no matter how simply designed. However, a single color dimension code showed good acceptance and a successful decoding rate, making this kind of coding mechanism affordable to this community for subtle assisted interaction.

In particular, we assumed a-priori that the guessed number of days in C2 would be more accurate than that in C1, since C2 had four categories instead of three, and therefore each of them codified a narrower range of possible days to guess from. However, the analysis revealed the opposite, showing that the better precision in right guesses in C2 was clouded by an increase in the number of wrong guesses due to the extra difficulty of identifying concepts in a multidimensional code.

## 6. Conclusions

Human to human interaction is a multi-level dynamic process for which current technology presents many gaps, limiting communication and interaction to their most explicit and demanding ways such as phone calls, video conferences or e-mails. These gaps affect particularly to people with special needs trying to live an independent life which are forced to either deal with strong intrusions or long periods of isolation.

To fill in these gaps we have focused on the importance of subtle communication to strengthen the social fabric, as it works at an empathy level based on common knowledge and experiences; and of soft communication to provide unobtrusive guidance.

We have presented a theoretical framework to define Subtle Interaction combining the interaction categorization of Ju and Leifer [18] with a reaction-aware classification of communication inspired by Shannon's definition of mutual information [19].

Based on this framework we have presented a theoretical analysis of the parts and pieces involved in Subtle Interaction to further design technologies to remotely support it.

We have suggested an Augmented Objects prototype to take advantage of a distributed interface to implicitly codify part the message. Additionally, we have defined, through a series of experiments, a rough limit for short memory in person-color associations and some color and symbol natural languages for expressing “urge” or “negativity”.

The limitations of the color-coded language have been tested within a population with cognitive disabilities showing that, for a small set of concepts, a color association poses little challenge, significantly increasing the response time of heavier traffic alternatives such as counting blocks.

In addition, the impact of introducing multiple dimensions on a code (theoretically increasing its granularity and expression capabilities) has also been studied within a population with cognitive disabilities, finding that multidimensionality poses a great challenge no matter how simply it is designed.

Nevertheless, our results suggest that further studies must be conducted to identify variations among sub-populations, studying in depth the different etiologies of cognitive disabilities and the specific difficulties they face (language/communication, daily living tasks, following instructions, *etc.*). In this direction, we are planing to extend the evaluation to people with cognitive decline (older adults), a group with less willingness to adopt new technologies but in which better cognitive results are to be expected.

We believe that while in general it is desirable to conceive and develop technologies to support the rich variety of types of communication an interaction we are accustomed to in face to face scenarios, these type of technologies will especially help people with special needs to live independently a socially complete life.

## Figures and Tables

**Table 1.** Color comparison.

	**Red (109)**	**Yellow (75)**	**Blue (44)**
Yellow (75)	−34 ***/7 – 37 ***	–	–
Blue (44)	−65 ***/8 – 36 ***	−31 ***/11 – 33 ***	–
Green (36)	−73 ***/8 – 36 ***	−39 ***/8 – 36 ***	−8/19 – 25

* *p* < 0.1

** *p* < 0.05

*** *p* < 0.001 using *χ*^2^ test.

**Table 2.** Symbol comparison.

	**Saltire × (181)**	**Dash - (140)**	**Cross + (115)**	**Square □ (76)**	**Triangle △ (76)**
Dash - (140)	−41 ***/13 – 31 ***	–	–	–	–
Cross + (115)	−66 ***/5 – 39 ***	−25 */17 – 27 *	–	–	–
Square □ (76)	−105 ***/7 – 37 ***	−64 ***/12 – 32 ***	−39 **/12 – 32 ***	–	–
Triangle △ (76)	−105 ***/5 – 39 ***	−64 ***/8 – 36 ***	−39 **/11 – 33 ***	**0/18 – 26**	–
Circle ○ (72)	−109 ***/9 – 35 ***	−68 ***/12 – 32 ***	−43 ***/16 – 28 **	**−4/17 – 27** *	**−4/18 – 26**

* *p* < 0.1

** *p* < 0.05

*** *p* < 0.001 using *χ*^2^ test.

**Table 3.** Wrong answers in condition 1.

**Answered concept**				

		FRESH	URGENT	EXPIRED
Correct	FRESH	-	0	0
concept	URGENT	0	-	3
	EXPIRED	0	4	-

**Table 4.** Wrong answers in condition 2.

**Answered concept**					

		FRESH	ACCEPTABLE	URGENT	EXPIRED
Correct	FRESH	-	1	1	0
	ACCEPTABLE	0	-	10	0
concept	URGENT	1	14	-	0
	EXPIRED	0	1	4	-
